# Postoperative fluid overload is a risk factor for adverse surgical outcome in patients undergoing esophagectomy for esophageal cancer: a retrospective study in 335 patients

**DOI:** 10.1186/s12893-016-0203-9

**Published:** 2017-01-13

**Authors:** Torben Glatz, Birte Kulemann, Goran Marjanovic, Svenja Bregenzer, Frank Makowiec, Jens Hoeppner

**Affiliations:** Department of General and Visceral Surgery, Medical Center - University of Freiburg and Faculty of Medicine - University of Freiburg, Hugstetter Str. 55, D-79106 Freiburg, Germany

**Keywords:** Esophageal cancer, Esophagectomy, Perioperative fluid management, Morbidity, Adverse surgical outcome

## Abstract

**Background:**

Restrictive intraoperative fluid management is increasingly recommended for patients undergoing esophagectomy. Controversy still exists about the impact of postoperative fluid management on perioperative outcome.

**Methods:**

We retrospectively examined 335 patients who had undergone esophagectomy for esophageal cancer at the University Hospital Freiburg between 1996 and 2014 to investigate the relation between intra- and postoperative fluid management and postoperative morbidity after esophagectomy.

**Results:**

Perioperative morbidity was 75%, the in-hospital mortality 8%. A fluid balance above average on the operation day was strongly associated with a higher rate of postoperative mortality (21% vs 3%, *p* < 0.001) and morbidity (83% vs 66%, *p* = 0.001). Univariate analysis for risk factors for adverse surgical outcome (Clavien ≥ III) identified ASA-score (*p* = 0.002), smoking (*p* = 0.036), reconstruction by colonic interposition (*p* = 0.036), cervical anastomosis (*p* = 0.017), blood transfusion (*p* = 0.038) and total fluid balance on the operation day and on POD 4 (*p* = 0.001) as risk factors.

Multivariate analysis confirmed only ASA-score (*p* = 0.001) and total fluid balance (*p* = 0.001) as independent predictors of adverse surgical outcome.

**Conclusion:**

Intra- and postoperative fluid overload is strongly associated with increased postoperative morbidity. Our results suggest restrictive intra- and especially postoperative fluid management to optimize the outcome after esophagectomy.

## Background

Surgery for esophageal cancer has traditionally been associated with high postoperative morbidity and mortality and poor long-term survival, but results have improved dramatically over the last decade [1, 2]. Improvements in perioperative management and surgical technique have contributed to a reduced rate of surgical and medical complications, but the impact of each individual factor remains vague.

Among other factors, restrictive intraoperative fluid management has proven to reduce perioperative morbidity in colorectal and pancreatic surgery [3]. While nowadays restrictive intraoperative fluid management is accepted as favourable in esophageal surgery as well as in other major abdominal operations [4], very little evidence exists supporting this regimen, and even less concerning the early postoperative phase. Small studies were able to establish a correlation between intraoperative fluid overload and increased postoperative pulmonary morbidity as well as an increased number of surgical complications after esophageal surgery [5–7]. These studies are however limited due to very small numbers of patients, their retrospective study design and short observation periods.

To date no study provides sufficient evidence supporting restrictive intra- and postoperative fluid management after esophagectomy. The aim of this study was thus to evaluate risk factors for surgical and pulmonary morbidity focusing on the impact of intraoperative and especially postoperative fluid management.

## Methods

This study evaluates the outcome of 335 consecutive patients with esophageal cancer undergoing esophagectomy between 01/1996 and 03/2014 at a high-volume tertiary referral centre. Our primary Hypothesis was that restrictive postoperative fluid management is associated with a reduced postoperative morbidity. Informed consent was obtained from all patients before their inclusion in the cancer registry. The Medical Ethics Committee of the University of Freiburg approved the study.

### Pretherapeutic work-up and multimodal treatment

Diagnostic work-up included endoscopy with biopsies and thoraco-abdominal computerized tomography (CT) in all patients, as well as cardiac and pulmonary work-up. Endoscopic ultrasound was used routinely for locoregional staging if technically possible. In general, lymph nodes were preoperatively classified as malignant if >1 cm by computerized tomography or endoscopic ultrasound. PET-CT was reserved for diagnostic nondestinctive cases.

Neoadjuvant chemoradiation has been performed since 1994 when (after initial staging) the T stage was T3 or T4 and/or lymph nodes were suspected to be positive, and patients had no other medical contraindication for neoadjuvant chemoradiation. Neoadjuvant chemoradiation was performed according to a protocol suggested by Naunheim et al. [8] or the CROSS protocol [9]. Radiation dose was increased from 36 GY to 45 Gy after 2011.

Neoadjuvant chemotherapy has been performed since 2006 in patients with Adenocarcinoma (AC) of the distal esophagus and esophagogastric junction if the T stage was T3 or T4 and/or lymph nodes were suspected to be positive, and patients had no other medical contraindication for neoadjuvant chemotherapy. Neoadjuvant chemotherapy was performed according to the protocol suggested by Cunningham et al. [10] or the FLOT protocol [11]. All neoadjuvant chemotherapy protocols were scheduled for postoperative continuance starting 4–8 weeks after the operation with the same drug composition and dosage as preoperatively.

After neoadjuvant chemoradiation or neoadjuvant chemotherapy, the patients were restaged by endoscopy and CT, and resection was performed approximately 6 weeks after the end of neoadjuvant treatment.

### Surgery

The operative procedure was chosen according to tumor location. Patients were mostly operated by an Ivor-Lewis thoraco-abdominal approach (right-sided thoracotomy, median laparotomy, collar hand-sewn or intrathoracic stapled anastomosis), a few underwent esophagectomy by a transmediastinal approach (laparotomy, no thoracotomy, collar approach with hand-sewn anastomosis). Reconstruction was routinely performed by a gastric tube formation and pull-up, continuity was established only in individual cases by a colonic interposition. We routinely performed two-field lymphadenectomy in patients with a thoraco-abdominal approach. In patients with a transmediastinal approach, lymphadenectomy was limited to the lower mediastinum and abdominal compartment. The majority of patients received an intraoperatively placed jejunostomy catheter for postoperative enteral feeding.

### Perioperative management

Neither the anesthetic approach, nor postoperative care processes were strictly standardized at our institution. Whenever possible, patients received an epidural catheter for intra-and postoperative pain management. Patients were kept on a surgical intermediate care unit for at least 4 days after surgery. Patients were restricted to oral fluids until postoperative day (POD) 5 and started on solids after a routine contrast esophagogram ruled out anastomotic leak or stenosis. Patients usually received 1ml/kg/h of Normofundin ® G-5 solution [Braun, Melsungen, Germany] on the day of operation after admission to the intensive care unit. Enteral feeding via jejunostomy catheter was started 6 h after surgery and was continued till patients were able to consume sufficient solid food. Patients without a jejunostomy catheter received total parenteral nutrition instead. In the operating room and on the intermediate care unit, all patients were monitored according to standard parameters regarding blood pressure (systolic pressure goal >90 mmHg), and urine output (targeting 0.3–0.5 ml/kg/h). Noradrenalin was the standard catecholamine used intra- and postoperatively in almost all cases.

Blood product transfusions were generally initiated when hemoglobin levels were less than 8 g/dl in patients without coronary artery disease or less than 10 g/dl in patients with coronary artery disease. The crystalloid fluid used was a balanced electrolyte solution (either Jonosteril® [Fresenius Kabi, Bad Homburg Germany] or Normofundin® G-5 [Braun, Melsungen, Germany]). The colloidal solutions were based on hydroxyethyl starch (HES) 6% (either Voluven® or Volultye® [Fresenius Kabi, Bad Homburg, Germany]) with less sodium and chloride and an acetate buffer.

### Assessment of perioperative fluid management and surgical outcome

Intraoperative fluid administration was extracted from the anesthetic protocols, the postoperative amount of administered fluid from the electronic health records of each patient. The type and amount of fluids given during and after esophagectomy was evaluated. In further detail, the amount and type of fluids administered during the operation was assessed as well as the sum (of each type) of fluids given on the operation day and until the end of POD 4. Thus, not only the specific amount of fluids given during the operation, but also the fluids given postoperatively on the intensive care unit are displayed and evaluated. Not considered were perspiratio insensibilis and additional fluid loss not recorded in the charts. Perioperative blood transfusion was defined as any number of erythrocyte concentrates administered intraoperatively or until POD 4.

Furthermore, the correlation of the intra- and postoperative fluid administration and surgical outcome was investigated. Postoperative complications were noted and graded according to the Clavien-Dindo classification [12]. Primary endpoint of our study was adverse surgical outcome, which was defined as at least one postoperative complication graded Clavien-Dindo 3 or higher, thus requiring any endoscopic, surgical or radiological intervention. Complications contributing to this endpoint were anastomotic leak, delayed gastric emptying, intra-abdominal abscess, hemorrhage, wound infection, cardiac and respiratory complications. After discharge from hospital, patients were followed up at the surgical outpatient department and referred back either to the department of medical oncology or to a resident oncologist for adjuvant therapy (when indicated) and further follow-up. Complications were recorded up to 3 months after discharge.

### Statistical analysis

The results of our study were gained by retrospective analysis of our prospectively maintained esophagogastric database. IBM SPSS Statistics for Windows, (Version 21.0 Armonk, NY USA: IBM Corp.) was used for statistical analysis. Categorical variables were put in absolute and relative frequencies; differences were evaluated by one-tailed Fisher’s exact test. Quantitative values were expressed as medians with range, and differences were measured using the Mann-Whitney-*U*-test or Kruskal-Wallis-test as appropriate. Multivariate logistic regression analysis with forward stepwise including was used to identify independent risk factors for adverse surgical outcome. Because overlapping variables (Fluid balance on POD 0 and POD 4) were used in our analyses, multivariate analysis was performed in different models with the inclusion of only one of these variables each time to prevent multicollinearity. Inclusion *p* for multivariate analysis was 0.10. A *p*-value <0.05 was considered statistically significant.

## Results

### Patients demographics & tumor and treatment characteristics

Between 1996 and 2014, 335 patients underwent esophagectomy at our institution for esophageal cancer. Eighty-seven percent of the patients were male and the mean age was 62 years. The majority of tumors were adenocarcinomas (59%) and located in the lower third of the esophagus (75%). Neoadjuvant chemoradiation was performed prior to the operation in the majority of cases (56%), while 22% received neoadjuvant chemotherapy and 22% unimodal surgical treatment (Table 1).

**Table 1:** Demographics. tumor and treatment characteristics

	*n* = 335
Male sex	290 (87%)
Age (yrs)	62 [30 - 90]
BMI (kg/m^2^)	24.5 [14.6 – 45.9]
ASA score	
1	10 (3%)
2	183 (55%)
3	137 (41%)
4	5 (2%)
Multimodal therapy	
None	73 (22%)
Neoadjuvant radiochemotherapy	189 (56%)
Perioperative chemotherapy	73 (22%)
Tumor localisation	
Upper third	14 (4%)
Middle third	72 (22%)
Lower third/junctional tumor	249 (74 %)
Surgical approach	
Thoracoabdominal	309 (92%)
Transhiatal	26 (8%)
Reconstruction	
Gastric tube	321 (96%)
Colon interposition	14 (4%)
Anastomosis	
Intrathoracic	185 (55%)
Cervical	150 (45%)
Tumor histological type	
Squamous cell carcinoma	137 (41%)
Adenocarcinoma	198 (59%)
UICC-Stage	
0/1	111 (33%)
2	140 (42%)
3	63 (19%)
4	21 (6%)
Resection margin	
R0	316 (94%)
R+	19 (6%)
Median operating time (min)	420 [203 - 906]
Median ICU stay (days)	8 [1 - 121]
Median Hospital stay (days)	22 [3 - 150]

Displayed are demographic, tumour and treatment characteristics of 335 patients undergoing esophaegctomy

Ninety-two percent of the patients were operated by a thoraco-abdominal approach, the remaining 8% underwent esophagectomy by a transmediastinal approach. The anastomosis was located cervical in 45% and intrathoracic in 55% of the cases. In 96% reconstruction was performed by a gastric tube pull-up, and in the remaining 4% continuity was established by colon interposition. Median operating time was 420 min and median hospital stay 22 days.

Tumors were postoperatively staged according to the UICC-classification as Stage 0/I in 33% (including complete remission), Stage II in 42%, Stage III in 19% and Stage IV in 6%. Nineteen patients (6%) had a margin-positive resection (Table 1).

### Intra- and postoperative fluid balance

The intra- and postoperative fluid management is displayed in Table 2.

**Table 2:**
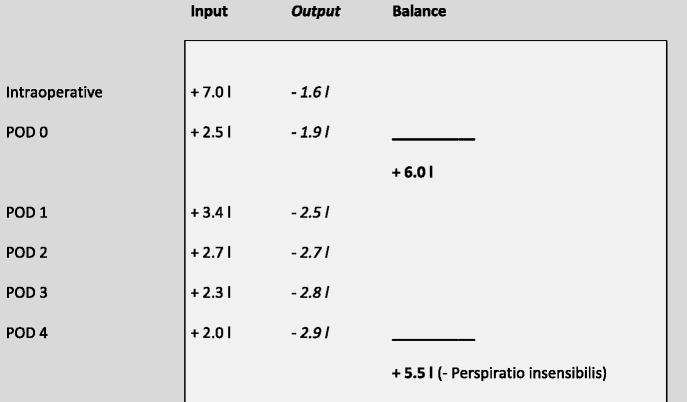
Intra- and perioperative fluid balance

The table shows the intraoperative and postoperative fluid intake (intravenous fluids, transfusions, nutrion) and output (blood loss, drains, urin) on POD 0-4 including and the total balance resulting on POD 0 and 4

**Fig. 1:**
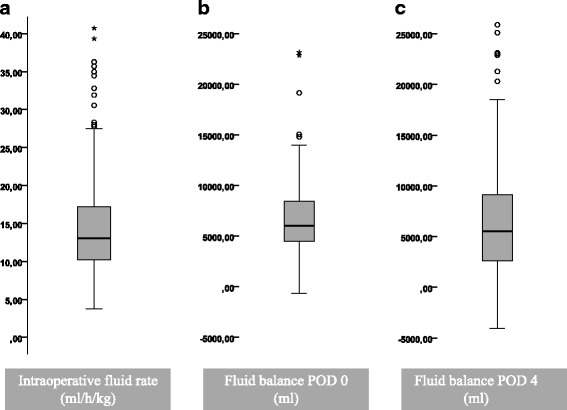
Box-Whisker-Plot displaying the introperative and postoperative fluid balance. The median intraoperative fluid intake was 13 ml/kg/h (**a**), the fluid balance added up to a median of 6000 ml on POD 0 (**b**) and a median of 5500 ml POD 4 (**c**). 76.9% of patients were identical in the groups on POD 0 and POD 4

From our data, we calculated a total median fluid overload of 6000 ml (range 600 – 24300 ml) at the end of the operation day (POD 0) and 5500 ml (range 4000 – 37200 ml) at the end of POD 4. The actual fluid balance has to be estimated lower considering the additional fluid loss like perspiratio insensibilis, which would be very hard to quantify. Data regarding fluid input and output were not available for 2 patients intraoperatively, one additional patient on the operation day and for another three additional patients for POD 1 – 4. Patients were divided into groups according to their fluid balance on POD 0 and again on POD 4: Fluid balance above the median was defined as fluid overload, below median as fluid restrictive. The majority of patients (77%) were identical in the fluid groups on POD 0 and POD 4, but 11% with intraoperative fluid overload were below average on POD 4, while 12% with restrictive fluid management during the operation showed an above-average fluid balance on POD 4. Figure 1 displays the median IOF rate and total fluid balance on POD 1 and 4.

Erythrocyte concentrates were administered intraoperatively in 36% of the cases (*n* = 122) with a median amount of 900 ml (range 300 – 16200 ml), while fresh frozen plasma was given in only 12% of the cases (*n* = 40, median amount 700 ml, Range 200 – 11200 ml).

Until POD 4, 203 patients received erythrocyte concentrates (61%), administered was a median of 900 ml (range 300 – 16200 ml). Two hundred four patients received at least one unit of fresh frozen plasma (61%, median 1200 ml, range 200 – 9000). Sixty-seven percent of all patients required catecholamine therapy during the operation and directly after, while only 52% needed further catecholamine therapy in the postoperative phase. During the first four postoperative days, 61% of the patients received diuretics.

Patients operated before 2006 received significantly more intraoperative fluids (16 ml/kg/h versus 11 ml/kg/h 2006 and after, *p* < 0.001) resulting in a more distinct volume overload (8000 ml versus 5000 ml on POD and 8838 ml versus 3100 ml on POD 4, *p* < 0.001).

### Perioperative outcome and association with fluid balance

Seventy-five percent of our patients developed at least one postoperative complication according to the Clavien-Dindo-Classification [12]. Twenty-two percent were managed conservatively (Grade II), while 44% of the patients required further intervention or operation (Grade III/IV). The in-hospital mortality was 8% (Grade V). Most complications were either of pulmonary (in 53%) or surgical origin (in 47%). Twenty five percent of the patients developed a medical complication, mostly cardiac incidents (20%) or renal failure (15%). Table 3 shows the frequency and severity of postoperative complications and the association with fluid overload on POD 0 and 4. It is of note, that a fluid balance above average on the operation day was strongly associated with a higher rate of postoperative mortality (13 % vs 3%, *p* < 0.001) and complications (83% vs 66%, *p* = 0.001), especially severe complications (Grade III or higher). Higher rates of pneumonia, pleural effusion, need for tracheostomy, anastomotic leakage, and even cardiac incidents were observed. Interestingly, pulmonary embolism was the only complication registered almost exclusively in the group with restricted fluid balance (8 cases vs 1 case, *p* = 0.018).

**Table 3:** Association of intra- and postoperative fluid management with postoperative morbidity

		Fluid balance POD 0 (ml)			Fluid balance POD 4 (ml)		
Parameter	Total	<6000	>6000	p^a^	<5500	>5500	p^a^
	*n* = 333	*n* = 166	*n* = 166		*n* = 164	*n* = 165	
In-hospital mortality	26 (8%)	5 (3%)	21 (13%)	*<0.001*	2 (1%)	22 (13%)	*<0.001*
Perioperative morbidity	248 (75%)	110 (66%)	137 (83%)	*0.001*	107 (65%)	137 (83%)	*<0.001*
Pulmonary morbidity	175 (53%)	79 (48%)	97 (58%)	*0.031*	74 (45%)	100 (60%)	*0.003*
Pneumonia	112 (34%)	42 (25%)	72 (43%)	*<0.001*	42 (26%)	71 (43%)	*0.001*
Pleural effusion	102 (31%)	44 (27%)	59 (36%)	*0.048*	41 (25%)	60 (36%)	*0.017*
Re-intubation	64 (19%)	27 (16%)	39 (24%)	0.065	20 (12%)	44 (27%)	*0.001*
Tracheostomy	38 (11%)	14 (8%)	25 (15%)	*0.044*	9 (6%)	30 (18%)	*<0.001*
Pulmonary embolism	9 (3%)	8 (5%)	1 (1%)	*0.018*	9 (6%)	0 (0%)	*0.002*
Surgical morbidity	156 (47%)	62 (37%)	93 (56%)	*<0.001*	60 (37%)	94 (57%)	*<0.001*
Wound infection	60 (18%)	23 (14%)	35 (21%)	0.056	20 (12%)	38 (23%)	*0.007*
Anastomotic leakage	49 (15%)	15 (9%)	34 (21%)	*0.002*	12 (7%)	37 (22%)	*<0.001*
Other	82 (25%)	33 (20%)	49 (30%)	*0.028*	30 (18%)	50 (30%)	*0.008*
Cardiac Complications	66 (20%)	26 (16%)	40 (24%)	*0.037*	23 (14%)	41 (25%)	*0.009*
Renal Complications	15 (5%)	5 (3%)	10 (6%)	0.145	2 (1%)	11 (7%)	*0.010*
Severity of complications^b^				*<0.001*			*<0.001*
Grade 0/I	85 (25%)	56 (34%)	29 (18%)		57 (35%)	28 (17%)	
Grade II	76 (22%)	39 (24%)	37 (22%)		38 (23%)	38 (23%)	
Grade IIIa/b	97 (29%)	42 (25%)	54 (32%)		48 (29%)	47 (29%)	
Grade IVa/b	49 (15%)	24 (15%)	25 (15%)		19 (12%)	30 (18%)	
Grade V	26 (8%)	5 (3%)	21 (13%)		2 (1%)	22 (13%)	

^a^Fisher’s exact test ^b^Complications were graded according to Clavien/Dindo

Patients were divided by their total fluid balance (median cut) on POD 0 and POD 4. The table compares the postoperative morbidity and mortality rate in the groups and the occurrence of specific complications

Statistical analysis showed an even more distinct correlation for the above-average fluid balance on POD 4, with significant values even for the rate of reintubations (*p* < 0.001), wound infection (*p* = 0.007) and renal complications (*p* = 0.01). Not surprisingly, neither fluid balance on POD 0 nor POD 4 had any influence on the rates of postoperative pneumo- and chylothorax, pleural empyema, anastomotic stricture, postoperative ileus and deep vein thrombosis.

**Fig. 2:**
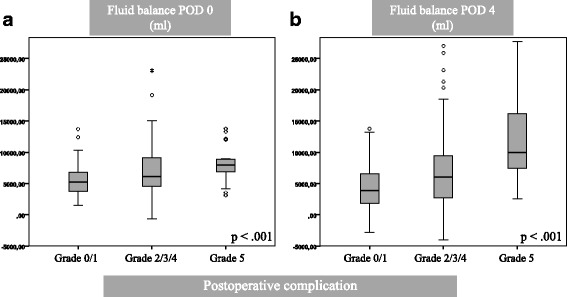
Box-Whisker-Plot displaying the correlation between the median fluid balance on POD 0 (**a**) and POD 4 (**b**) and the severity of postoperative complications. Complications were graded according to Clavien/Dindo. The median fluid balance was significantly higher in patients with postoperative complications on POD 0 as well as on POD 4

The total fluid balance on POD 0 and 4 correlated significantly with the severity of postoperative complications: Patients with no complication had a median fluid balance of 5300 ml on POD 0 and 3900 ml on POD 4, while patients with a complication Grade II-IV had a fluid overload of 6100 ml on POD 0 and 6000 ml on POD 4. Patients who died subsequent to the operation had received even higher amounts with 8000 ml on POD 0 and 10000 ml on POD 4 (*p* < 0.001). Figure 2 shows the correlation between the severity of postoperative complications and the fluid balance on POD 0 and 4.

### Risk factor analysis for adverse surgical outcome

Patients with a complication grade III or higher were defined as having an adverse surgical outcome (52%) and underwent further risk factor analysis for adverse surgical outcome. Univariate analysis (Table 4) identified ASA-score (*p* = 0.002), recent smoking (*p* = 0.036), reconstruction by colonic interposition (*p* = 0.036), cervical anastomosis (*p* = 0.017) and perioperative blood transfusion (*p* = 0.038) as risk factors. Sex, age, BMI, alcohol abuse, type of multimodal therapy, surgical approach, tumour localisation, histological type and stage were not associated with adverse surgical outcome. Most importantly, both, total fluid balance on the operation day as well as on POD 4 were strongly associated with adverse surgical outcome (*p* = 0.001), while the sole rate of intraoperative fluid administration could not be identified as a risk factor (*p* = 0.479).

**Table 4:** Univariate analysis of predictors for adverse surgical outcome

Parameter	Number	Adverse surgical outcome^a^	P^b^
Total	335	174 (52%)	
Sex (*n*, %)			0.389
Male	290	152 (53%)	
Female	45	22 (49%)	
ASA score (*n*, %)			*0.002*
1 – 2	193	87 (45%)	
3 – 4	142	87 (61%)	
BMI			0.252
<25 kg/m^2^	179	96 (53%)	
≥25 kg/m2	154	76 (49%)	
Recent Smoking (*n*, %)			*0.036*
Yes	170	97 (57%)	
None	165	77 (47%)	
Multimodal therapy (*n*, %)			0.081
None	73	43 (59%)	
Neoadjuvant radiochemotherapy	189	101 (53%)	
Perioperative chemotherapy	73	30 (41%)	
Surgical approach (*n*, %)			0.342
Thoracoabdominal	309	159 (52%)	
Transhiatal	26	15 (58%)	
Reconstruction (*n*, %)			*0.036*
Gastric tube	321	163 (51%)	
Colon interposition	14	11 (79%)	
Anastomosis (*n*, %)			*0.017*
Intrathoracic	185	88 (59%)	
Cervical	150	86 (47%)	
Tumor histological type (*n*, %)			0.383
Squamous cell carcinoma	137	73 (53%)	
Adenocarcinoma	198	101 (51%)	
UICC-Stage (*n*, %)			0.149
1 – 2	251	135 (54%)	
3 – 4	84	39 (46%)	
Treatment period			*0.015*
1996 – 2005	159	93 (53%)	
2006 – 2014	176	81 (46%)	
Perioperative blood transfusion			*0.038*
Yes	203	112 (55%)	
No	126	56 (44%)	
Intraoperative fluid rate			0.479
<13 ml/kg/h	166	85 (51%)	
≥13 ml/kg/h	167	87 (52%)	
Fluid balance POD 0			*0.001*
<6000 ml	166	71 (43%)	
≥6000 ml	166	100 (61%)	
Fluid balance POD 4			*0.001*
<5500 ml	164	69 (42%)	
≥5500 ml	165	99 (60%)	

^a^At least one Complication grade 3 or higher graded according to Clavien/Dindo, ^b^Fisher's exact test

We performed a risk factors analysis for the occurrence of adverse surgical outcome (defined as at least one complication graded 3 or higher). Displayed are the analysed risk factors and their impact on the outcome

Multivariate analysis (Table 5) confirmed only ASA-score (*p* = 0.001) and fluid balance on POD 0 (*p* = 0.001) as independent predictors of adverse surgical outcome. Fluid balance on POD 4 was analysed in a separate model to prevent multicollinearity and was likewise confirmed as an independent risk factor(*p* = 0.001).

**Table 5:** Multivariate analysis of predictors for adverse surgical outcome^a^

Predictor	OR	CI	p^b^
Recent Smoking (Y/N)	---	---	0.162
Multimodal therapy (RCTX/CTX)	---	---	0.415
Anastomosis (cervical/intrathoracic)	---	---	0.741
Reconstruction (Colon/gastric tube)	---	---	0.091
Perioperative blood transfusion (Y/N)	---	---	0.617
Treatment period (1996-2005/2006-14)	---	---	0.741
ASA score (3-4/1-2)	2.12	1.34 - 3.35	*0.001*
Fluid balance POD 0 (≥/< 6000 ml)	2.03	1.29 - 3.19	*0.001*
Fluid balance POD 4 (≥/< 5500 ml)	2.13	1.35 - 3.34	*0.001*

^a^At least one Complication grade 3 or higher graded according to Clavien/Dindo

^b^Multivariate logistic regression analysis with forward stepwise including, Odds ratio (OR), 95% confidence interval (CI)

The table shows the multivariate analysis of the risk factors identified by the univariate analysis. Fluid balance on POD 0 and POD 4 were analysed in separate models to prevent multicollinearity

We noticed a shift of fluid management towards a more restrictive regime over time. To analyse the correlation between year of treatment and postoperative morbidity, patients were divided by treatment period in two groups (1996–2005 and 2006–14). Patients treated after 2006 had a significantly lower postoperative morbidity (46% vs 53%, *p* = 0.015). In multivariate analysis treatment period was eliminated (*p* = 0.741). Therefore the effect has to be attributed to the changes made in perioperative management and surgical technique.

## Discussion

Our single-center retrospective study analyses 335 patients undergoing esophagectomy for esophageal cancer represents and is the largest cohort study ever on intra- and postoperative fluid management and its impact on perioperative outcome of esophagectomy.

Postoperative morbidity was high with over 50% of patients developing a relevant complication and with 8% in-hospital mortality. However, these results are consistent with perioperative outcome described in the recent literature [13–15]. Our data suggest that intra- and postoperative fluid restriction is associated with an improved perioperative outcome, matching the results of several investigations from the field of pancreatic and colorectal surgery [3].

Few observations also suggest restrictive fluid management for patients undergoing esophagectomy [4, 16, 17]. To date, this recommendation is based on very limited evidence. Early case series applying restrictive fluid management to patients undergoing esophagectomy show favourable results regarding postoperative morbidity, but none of these studies was randomized [16, 17]. Retrospective analysis in small cohorts of patients were able to show a correlation between intraoperative fluid overload and adverse surgical outcome in patients after esophagectomy [6, 18] and especially an association between fluid overload and pulmonary morbidity after Ivor-Lewis-esophagectomy [5]. One very small randomized controlled trial with 22 patients failed to establish this correlation. Improved pulmonary function was demonstrated after restrictive fluid management, but no effect on postoperative complications [7].

Even though restrictive fluid management nowadays is considered beneficial by most surgeons, it remains hard to achieve. Our study shows a decrease of intra- and postoperative fluid overload over time. Strategies used to optimize postoperative fluid balance are the restriction of intravenous fluids, the selective use of catecholamin therapy and the generous application of diurectics starting as early as possible. Another possibility to direct fluid management are goal-directed strategies. Goal-directed and “restrictive” intraoperative fluid management strategies have been established in different surgical specialties (pancreatic and colorectal) and are supported by sufficient evidence provided by randomized trials and systemic reviews [3]. These strategies have also been transferred to esophageal surgery, although supporting data for this specific field are scarce [19]. Our results show that the sole quantification of fluid input is not associated with impaired outcome, but that total fluid balance is. These results would support goal-directed therapy as a guideline for fluid management.

The pathophysiology of our findings may mainly be related to fluid impact on perioperative lung physiology. The postoperative morbidity of esophagectomy strongly consists of the pulmonary component [5]. Restrictive fluid administration has proven to prevent acute lung injury in patients after lung resection [20], a mechanism that might be transferred to esophagectomy. In our cohort, pulmonary complications occurred significantly more often in patients with intraoperative (58% vs 48%) or postoperative fluid overload (60% vs 45%).

Impaired wound healing may also contribute to morbidity and mortality after esophagectomy. In experimental settings, intraoperative fluid overload impairs gastrointestinal wound healing [21–24], another mechanism that might explain improved outcome related to restrictive fluid management in our study: Intraoperative and postoperative fluid overload were associated with a threefold incidence of anastomotic leakage in our analysis.

Our study analyses fluid administration over 4 postoperative days and correlates the results with perioperative outcome. Most other previous studies focused on intraoperative fluid administration. Postoperative fluid management even has been analyzed over the course of 2 days by only one study [6]. Our results show that fluid overload in the postoperative phase is equal to intraoperative fluid overload as a risk factor for postoperative morbidity. It can be assumed that complications like pneumonia and anastomotic leakage will contribute additionally to fluid overload due to impaired cardiac circulation and capillary leak syndrome. Put into perspective, since these complications usually manifest clinically after POD 4 [25], and certainly not intraoperatively, we believe that this factor is largely negligible.

The retrospective design of our study certainly allows only limited conclusions. Confounding factors leading to intra- and postoperative fluid overload and increased morbidity and mortality can only be partially eliminated by multivariate analysis. Possibly this study overvalues the negative effect of intra- and postoperative fluid overload on the postoperative morbidity. Our analysis shows that intraoperative infusion have been reduced during the study period and that a restrictive fluid management has already been implemented in our hospital and is likely to be an essential contributor to the reduced postoperative morbidity after esophagectomy [1, 2]. On the other hand improvement of surgical techniques and perioperative management over time is another confounder than cannot be eliminated by this analysis.

A further limitation of our study is its non-randomized character. Patients were not randomized to restrictive or liberal fluid management, but the decision was made to some extent arbitrarily by the attending anesthesiologist and surgeon. Operating time and intraoperative blood loss might effect both postoperative fluid balanace and morbidity. Blood transfusions were given in 61% of the cases intra- or postoperatively and contributed to the observed fluid overload. Perioperative blood transfusion itself has been identified as a risk factor for adverse surgical outcome in other analyses [26, 27]. The negative effects of transfusion like immunosuppressive effects mingle with the effects of volume overload and are difficult to distinguish in our analysis. Multivariate analysis eliminates perioperative blood transfusion as a risk factor in our analysis in favor of volume overload. We believe, that volume overload has to be considered as a confounding factor in future analyses regarding intra- and postoperative blood transfusion.

## Conclusion

Our study identifies postoperative fluid overload alongside high ASA-score as the strongest risk factor for adverse surgical outcome after esophagectomy for esophageal cancer. In the context of previous studies from different surgical specialties our results suggest that a restrictive fluid management can contribute to a further reduction of postoperative complications after esophagectomy.
